# LTR retrotransposons and the evolution of dosage compensation in *Drosophila*

**DOI:** 10.1186/1471-2199-9-55

**Published:** 2008-06-04

**Authors:** Lilya V Matyunina, Nathan J Bowen, John F McDonald

**Affiliations:** 1 School of Biology and Petit Institute for Bioengineering and Bioscience, Georgia Institute of Technology, Atlanta, GA 30332, USA

## Abstract

**Background:**

Dosage compensation in *Drosophila *is the epigenetic process by which the expression of genes located on the single X-chromosome of males is elevated to equal the expression of X-linked genes in females where there are two copies of the X-chromosome. While epigenetic mechanisms are hypothesized to have evolved originally to silence transposable elements, a connection between transposable elements and the evolution of dosage compensation has yet to be demonstrated.

**Results:**

We show that transcription of the *Drosophila melanogaster copia *LTR (long terminal repeat) retrotransposon is significantly down regulated when in the hemizygous state. DNA digestion and chromatin immunoprecipitation (ChIP) analyses demonstrate that this down regulation is associated with changes in chromatin structure mediated by the histone acetyltransferase, MOF. MOF has previously been shown to play a central role in the *Drosophila *dosage compensation complex by binding to the hemizygous X-chromosome in males.

**Conclusion:**

Our results are consistent with the hypothesis that MOF originally functioned to silence retrotransposons and, over evolutionary time, was co-opted to play an essential role in dosage compensation in *Drosophila*.

## Background

Retrotransposons are a major component of the genomes of higher eukaryotes and have been identified as a significant source of loss-of-function and regulatory mutations [1]. Over evolutionary time host genomes have developed mechanisms to mitigate the mutational potential of retrotransposons by transcriptionally silencing or otherwise blocking their transpositional activity [2]. One of the primary mechanisms by which retrotransposons are transcriptionally silenced is by methylation and/or other epigenetic mechanisms. Indeed it has been hypothesized that most, if not all, epigenetic mechanisms originally evolved as a defense against retrotransposons and have subsequently been co-opted for other essential cellular functions [3,4].

Approximately 10% of the *Drosophila melanogaster *genome is comprised of retrotransposons, the majority of which are LTR retrotransposons. LTR retrotransposon insertions are a major source of mutations in *D. melanogaster *and are believed to have contributed significantly to genome evolution [5]. While histone acetylation and other epigenetic mechanisms are believed to play an essential role in dosage compensation and other vital functions in *D. melanogaster*, little is known about the role of these mechanisms in the regulation of retrotransposons in this species [e.g., [6-8]]. In this paper, we present genetic and molecular evidence that the histone acetyltransferase, MOF, is involved in the transcriptional repression of the *copia *and perhaps other families of LTR retrotransposons in *Drosophila*. Our findings are consistent with the hypothesis that MOF may have originally functioned to silence retrotransposons and was subsequently co-opted for its role in dosage compensation.

## Results

**Figure 1:**
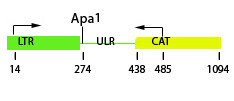
**Structure of the *copia *LTR-CAT construct**. Position of *Apa *I site (274 bp) and the 5' (14 bp) and 3' (485 bp) PCR primer binding sites are shown (LTR = 5' *copia *long terminal repeat; ULR= *copia *untranslated leader region; CAT= bacterial chloramphenicol acetyltransferase reporter gene (see Methods for primer sequences).

A series of *Drosophila melanogaster *strains transformed with a *copia *LTR-CAT (chloramphenicol acetyltransferase) reporter construct (Figure 1) was monitored for CAT activity in larvae and adult flies carrying the construct in the hemizygous vs. the homozygous state. Inconsistent with a simple additive model, CAT activity was found, on average, to be ~7-fold higher in strains homozygous vs. hemizygous for the construct (Table 1). In contrast, CAT activity of strains in which two copies of the hemizygous construct were present at non-homologous locations was consistent with an additive model (Table 2 and 3). Thus, the observed non-additive effect between hemizygous and homozygous *copia *elements is *cis*-dependent.

**Table 1:** Expression level of a *copia *LTR-CAT construct in nine stably transformed lines of *Drosophila melanogaster *made hemi- or homozygous for the construct

**Transformant**	**Insert Chrom-osomal Location**	-------------	**CAT ACTIVITY**	------------	---------------	**RATIO **(homo/hemi)
		**Female homozygous**	**Female hemizygous**	**Male homozygous**	**Male hemizygous**	

8-1	2L 40E	lethal	3.03 (0.33)			NA
"					lethal	2.9 (0.37)
9-1	2L 39A	lethal	0.32 (0.04)			
"					lethal	0.36 (0.06)
27-1	2R 46E	lethal	0.12 (0.03)			
"					lethal	0.11 (0.01)
9-3	3L 80A	3.82 (0.39)	1.50 (0.28)			2.55
"				4.01 (0.29)	0.98 (0.21)	4.09
9-4	2R 57B	2.27 (0.27)	0.22 (0.03)			10.31
"				2.42 (0.22)	0.21 (0.05)	11.52
9-6	3L 75C	3.60 (0.23)	0.56 (0.13)			6.43
"				3.65 (0.05)	0.32 (0.02)	11.41
14-2	4 102B	1.10 (0.35)	0.28 (0.03)			3.93
				1.26 (0.09)	0.26 (0.02)	4.85
14-1	× 16B	3.91 (0.10)	0.91 (0.09)			4.3
				NA	3.29 (0.04)	NA
39-2	× 5A	1.88 (0.13)	0.20 (0.04)			9.4
				NA	1.91 (0.24)	NA
						Av: 6.88

The results demonstrate a nearly seven-fold average increase in CAT activity in flies homozygous vs. hemizygous for the *copia *LTR-CAT construct

**Table 2:** Expression level of *copia *LTR-CAT in hemizygous *Drosophila melanogaster *flies.

Transformant	9-3	9-4	9-6	14-2
Hemizygous ♂	1.00 ± 0.14	0.32 ± 0.04	0.67 ± 0.08	0.22 ± 0.01
Hemizygous ♀	1.08 ± 0.21	0.32 ± 0.02	0.72 ± 0.02	0.31 ± 0.07

CAT activity of transformed *D. melanogaster *strains carrying a single hemizygous LTR-CAT construct inserted at various locations.

**Table 3:** Expression level of *copia *LTR-CAT in double hemizygous (2 hemizygous inserts in non-homologous chromosomal locations) of *Drosophila melanogaster *flies

**Transformant combination**	**Insert chromosomal locations**	**Male observed**	**Male expected**	**Female observed**	**Female expected**
9-3/9-4	3L 80A/2R 57B	1.32 ± 0.12	1.32	1.26 ± 0.09	1.41
9-3/9-6	3L 80A/3L 75C	1.77 ± 0.29	1.67	1.44 ± 0.19	1.81
9-3/14-2	3L 80A/4 102B	1.31 ± 0.18	1.24	1.09 ± 0.12	1.78
9-4/9-6	2R 57B/3L 75C	1.03 ± 0.21	0.99	1.06 ± 0.17	1.04
9-4/14-2	2R 57B/4 102B	0.66 ± 0.07	0.54	0.62 ± 0.04	0.63
9-6/14-2	3L 75C/4 102B	1.24 ± 0.13	0.99	1.29 ± 0.11	1.03

CAT activity of strains carrying two hemizygous copies of the LTR-CAT construct (see Additional file 1, Cross 1 for details of strain construction) are approximately equal to the values expected under the additive model (i.e., the sum of the CAT activities measured in two strains carrying single copies of the respective LTR-CAT constructs).

**Figure 2:**
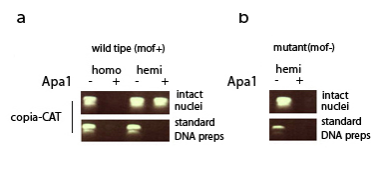
**PCR amplification products (*copia *LTR-CAT primers shown in Figure 1) of DNA prepared from intact nuclei (chromatin structure maintained) vs. DNA purified using standard procedures (chromatin structure not maintained-see Methods) digested (+) or not digested (-) with *Apa *I restriction enzyme**. (a) The results indicate that the *Apa *I restriction site is accessible for digestion in nuclei preps (chromatin structure preserved) from wild-type larvae homozygous for the *copia *LTR-CAT construct but is not accessible for digestion in nuclei preps from larvae hemizygous for the construct in the wild-type genetic background. (b) The resistance of hemizygous copies of the *copia *LTR-CAT construct is lost in larvae homozygous for the *mof*^1 ^(loss-of-function) allele. All experiments were conducted with the 9-3 transformant strain. [wild type (*mof *+) = DNA from larvae carrying the wild-type allele at the *mof *locus; mutant (*mof *-) = DNA from larvae carrying the *mof*^*1 *^allele at the *mof *locus; homo = DNA isolated from larvae homozygous for the *copia*LTR-CAT construct; hemi = DNA isolated from larvae hemizygous for the *copia*LTR-CAT construct].

Previous studies in other experimental systems have demonstrated that transposable elements are often partially or totally repressed due to host-mediated modifications in chromatin structure [e.g., [9-11]]. To determine if the non-additive effect we observed in *copia *expression is also associated with alterations in chromatin structure, we tested the ability of the *Apa*I restriction enzyme to access and digest a site in the *copia *LTR-CAT construct in larval chromatin extracts [12]. The results indicate that hemizygous copies of the *copia *LTR-CAT construct are being down regulated due, at least in part, to a chromatin mediated effect (Figure 2).

A number of genes have been shown to regulate chromatin structure in *Drosophila *[e.g., [6,7,13,14]]. To determine if members of this class of genes are involved in repressing the expression of hemizygous copies of the *copia *LTR-CAT construct, we monitored CAT activity in larvae or adult flies hemizygous for the construct within a series of genetic backgrounds mutant for genes known to affect chromatin structure in *Drosophila *[13,14] [*LOW/+, HDAC 1*^*326*^*/+, HDAC 1*^*328*^*/+, E(z)*^*28*^*/+, E(z)*^*61*^*/+, Psc25/+, Sxl*^*fl *^*/+*, and *mof*^*1 *^(Table 4, see Additional files 1,2, Tables 1, 2, 3, 4), presence of extra Y chromosome or absence of Y chromosome (see Additional file 2, Table 5)]. With the exception of *mof*^*1*^, each of the alleles tested display a dominant mutant phenotype in heterozygotes and are (embryonic) lethal when homozygous. Males hemizygous for the mutant (X-linked) *mof*^*1*^allele develop to the 3^rd ^instar stage but fail to metamorphose and hatch. In contrast, the phenotype and viability of females homozygous for the mutant *mof*^*1 *^allele is unaffected.

**Table 4:** Expression levels of *copia *LTR-CAT in stably transformed strains of *Drosophila melanogaster *hemizygous for the construct

**GENE**	**FEMALE**		**MALE**	
	**wild type**	**mutant**	**wild type**	**mutant**
*Low*	0.85 (0.08)	0.73 (0.10)	0.62 (0.05)	0.64 (0.12)
*HDAC 1*^*326*^	1.60 (0.27)	0.97 (0.16)*	1.31 (0.31)	0.93 (0.17)
*HDAC 1*^*328*^	"	0.88 (0.15)*	"	0.96 (0.20)
*E(z)*^*61*^*(29 C)*	0.54 (0.09)	0.51 (0.09)	0.38 (0.09)	0.43 (0.09)
" *(25 C)*	"	0.42 (0.08)	"	0.38 (0.10)
" *(18 C)*	"	0.41 (0.07)	"	0.42 (0.12)
*E(z)*^*28*^*(29 C)*	"	0.34 (0.07)*	"	0.34 (0.07)
" *(25 C)*	"	0.40 (0.12)	"	0.33 (0.10)
" *(18 C)*	"	0.28 (0.06)*	"	0.29 (0.08)
*Psc*^*25*^	"	0.17 (0.03)*	"	0.23 (0.03)*
*Sxl*^*fl*^	0.39 (0.26)	0.55 (0.12)	0.83 (0.12)	0.79 (0.14)
*mof*^*1*^	NA	NA	0.79 (0.07)	3.21 (0.35)**

*copia *LTR-CAT in a transformed strain (9-3) made wild type or heterozygous for dominant mutant alleles of genes known to affect chromatin structure [*LOW-lightening of white*; *HDAC 1^*326*^, HDAC 1^*328 *^-Histone Deacetylase; E(z)^*61 *^, E(z)^*28*^-Enhancer of zeste; Psc^*25*^-Posterior sex combs; Sxl^*fl*^-Sex lethal or *hemizygous for the X-linked recessive *mof*^*1 *^(males absent on first) allele. A slight but significant decrease in activity was observed in strains heterozygous for the dominant mutant *HDAC 1^*326 *^, HDAC 1^*328 *^, E(z)^*28 *^*and *Psc*^*25 *^alleles. A highly significant increase in activity was observed in the strain hemizygous for the recessive mutant *mof*^*1 *^allele

**Table 5:** Expression levels of *copia *LTR-CAT in stably transformed strains of *Drosophila melanogaster *hemizygous for the construct

**STRAIN**	***mof *+ hemizygous *copia *LTR-CAT**	***mof *– hemizygous *copia *LTR-CAT**
9-3	0.79 (0.07)	3.21 (0.35)**
9-4	0.31 (0.05)	2.53 (0.61)**
9-6	0.72 (0.10)	3.97 (0.21)**
14 -2	0.28 (0.08)	1.35 (0.27)**

Expression levels of *copia *LTR-CAT in males made hemizygous for the construct in a series of transformed strains (*copia *LTR-CAT inserted at different chromosomal locations in each strain-see Table 1) in wild-type vs. *mof*^*1 *^genetic backgrounds. The results indicate a position independent repression of hemizygous copies of the *copia *LTR-CAT by the wild-type *mof *allele (*p < 0.01; **p < 0.001).

No significant effect on LTR-CAT expression was detected in genetic backgrounds heterozygous for the dominant mutant *LOW*, *E(z)*^*61 *^and *Sxl *^*fl *^alleles (Table 4; see Additional file 2, Tables 1,3 and 4). Flies heterozygous for the dominant mutant *HDAC 1*^*326*^, *HDAC 1*^*328*^, *E(z)*^*28 *^and *Psc*^*25 *^alleles and for the presence of an extra Y chromosome or absence of a Y chromosome displayed a slight but significant (p < 0.01) decrease in CAT activity relative to controls (Table 4, see Additional file 2, Tables 2, 3 and 5). In contrast, a highly significant (p < 0.001) increase in the expression of the *copia *LTR-CAT constructs was observed in male larvae mutant for the recessive (hemizygous) X-linked *mof *^*1 *^allele (*males absent on first*) (Table 5). This increase in expression in the mutant *mof *^*1 *^background was observed in all of the independent *copia *LTR-CAT transformants examined (Table 5). Thus, the effect is not dependent upon the chromosomal location of the construct.

The protein product of the *mof *gene (MOF) is a member of the MYST family of histone acetyltransferases and, as part of the *Drosophila *MSL (male specific lethal) complex [15,16], has been shown to play an essential role in dosage compensation [e.g., [17-19]]. Our results are consistent with the effect of *mof *on *copia *LTR-CAT expression being chromatin-mediated (Figure 2).

**Figure 3:**
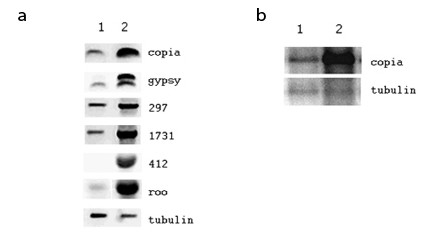
**(a). RT-PCR of mRNA isolated from 3rd instar larvae wild-type (column 1) or mutant (*mof*^*1*^) (column 2) at the *mof *locus using primers specific for five *Drosophila melanogaster *LTR retrotransposons (*copia, gypsy, 297, 1731, 412 and roo*) and β-*tubulin *as a control**. The results demonstrate uniformly higher levels of LTR retrotransposon expression in larvae carrying the mutant *mof*^*1 *^allele (see Methods for primer sequences). **(b). Northern hybridization of mRNA isolated from 3rd instar larvae wild-type (column 1) or mutant (*mof*^*1*^) (column 2) at the *mof *locus using *copia *[43] and β-*tubulin *[44] probes**. The results are consistent with RT PCR analyses (Figure 3a) and demonstrate higher levels of *copia *expression in flies carrying the mutant *mof*^*1 *^allele. No significant difference in β-*tubulin *expression was detected.

Since most active transposable elements (including LTR retrotransposons) are considered to be hemizygous in wild collected populations, we tested the effect of the mutant *mof *^*1 *^allele on global expression levels of *copia *and other families of LTR retrotransposons in a strain established from a natural population (Athens, GA). The results of a series of RT-PCR and Northern analyses presented in Figure 3 demonstrate that males carrying the mutant *mof *^*1 *^allele display a consistent and significant increase in the expression of all families of LTR retrotransposons examined. This suggests that the association of *mof *with down regulated expression of hemizygous copies of *copia *may extend generally to other families of LTR retrotransposons in *Drosophila*.

**Figure 4:**
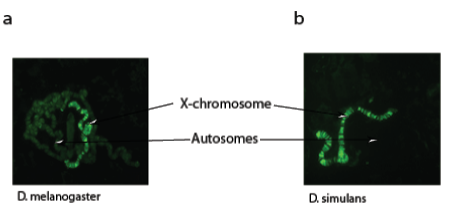
**Immunostaining of *Drosophila *polytene chromosomes with *MOF*-antibody**. Shown are preparations from strains representing two *Drosophila *species- (a) *D. melanogaster *and (b) *D. simulans*. Reduced binding of *MOF *to *D. simulans *autosomes is consistent with the reduced number of LTR retrotransposons present in this species.

While MOF is known to bind predominately to the male X-chromosome, weak binding also occurs at multiple locations on autosomes of *Drosophila melanogaster *[19] (Figure 4a). We observed a substantially reduced level of autosomal binding of MOF in *Drosophila simulans*, a species closely related to *melanogaster *but known to have significantly fewer numbers of LTR retrotransposons (Figure 4b) [20]. This suggests that at least some of the autosomal binding of MOF may be to LTR retrotransposon sequences.

To directly determine if MOF can bind to *copia *elements, we performed chromatin immunoprecipitation (ChIP) analyses using the *Drosophila melanogaster *Kc167 (female) cell line and previously described *Drosophila *MOF, MSL-1 and MSL-3 antibodies [21]. In males, these three proteins combine with MSL-2 (not expressed in females) and are sequestered to the X-chromosome as part of the MSL complex [21]. In females, MOF, MSL-1 and MSL-3 have been shown to be associated with all chromosomes at a reduced but significant level [19]. Since *copia *elements are known to be located on the X-chromosome, we chose to conduct our ChIP assays with the female Kc167 cell line in order not to confound *copia *specific binding by MOF with the more generalized X-chromosome binding that occurs in males. *Spt4 *is an autosomal gene previously shown not to be subject to dosage compensation [22]. *Rox *is a well-established binding site for MOF within the context of the MSL complex [23]. Since it has been shown that MSL-1, MSL-3 and MOF bind with reduced affinity to the X-chromosome in females, we selected *rox *as a putative positive control.

**Figure 5:**
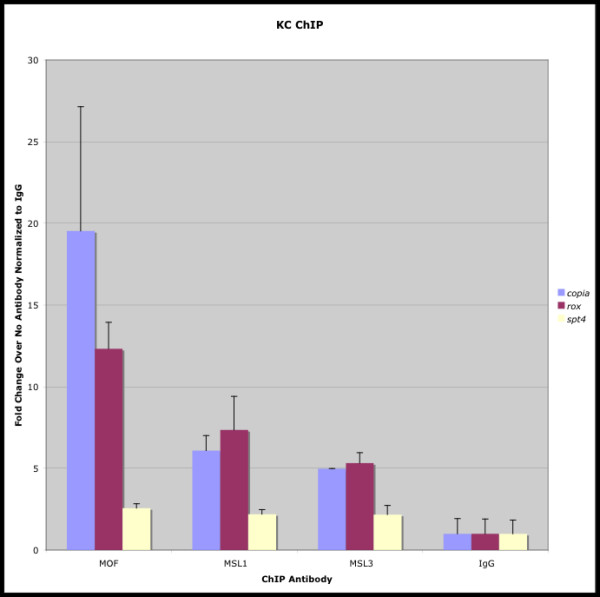
***MOF *is physically associated with the *copia *untranslated leader region (ULR) *in vivo***. Chromatin immunoprecipitation (ChIP) experiments were performed to determine whether *MOF *is physically associated with *copia *DNA sequences *in vivo*. Precipitation reactions performed independently with the *MOF*, the MSL-1 and MSL-3 antibodies, rabbit IgG antibody (non-specific control) and no antibody were used as templates for quantitative PCR from Kc cells. The average and standard errors for triplicate reactions were plotted to reveal the average fold increase in precipitated *copia *DNA for each antibody relative to the no antibody reactions. Precipitated DNA was amplified using primers specific for the *copia *ULR, the *roX-1 *gene (positive control) and the *Spt4 *gene (negative control) (see Methods). MOF and other members of the MSL complex have previously been demonstrated to bind to the *roX-*1 gene [e.g., 23]. *Spt-4 *was chosen as a negative control because it is not a target site for the MSL complex [e.g., 22]. The results indicate that MOF, MSL-1 and MSL-3 are all associated with the *copia *ULR and *roX-1 in vivo*. There was no binding above background to the *Spt-4 *gene.

The results presented in Figure 5 indicate that all three proteins bind to *copia *elements *in vivo *(and to a lesser extent to *rox*) but presumably not within the context of the MSL complex since MSL-2 is not expressed in females (Figure 5). Previous studies have demonstrated that the function of MOF within the context of the MSL complex is to acetylate lysine 16 on histone H4 (H4K16ac) [24], resulting in chromatin decondensation and increased transcription. In contrast, our results indicated that MOF is associated with reduced transcription of hemizygous copies of *copia *and perhaps other *Drosophila *LTR retrotransposons. Although the acetylation of histones is generally associated with transcriptional activation, it can also be involved in transcriptional repression [25-27]. Further studies will be required to determine the molecular basis of these findings.

## Discussion

A determination of the mechanistic basis of MOF mediated silencing of *copia *remains to be determined. Nevertheless, the fact that hemizygous copies of *copia *and perhaps other families of *Drosophila *LTR retrotransposons are the target of epigenetic repression, appears to be analogous to MSUD (meiotic silencing by unpaired DNA) in *Neurospora *where DNA unpaired in meiosis causes silencing of the unpaired sequence and all DNA homologous to it [28,29]. Similar phenomena have been reported in *C. elegans *[30,31] and mice [32], and have been associated with epigenetically mediated modifications in chromatin structure. Our results indicate that the repression of hemizygous copies of *copia *and other *Drosophila *LTR retrotransposons is also mediated by changes in chromatin structure. The fact that this repression appears to be mediated by MOF implies a relationship between retrotransposons and the evolution of dosage compensation in *Drosophila*.

While it is generally acknowledged that MOF plays an essential role in equalizing the expression of X-linked and autosomal genes in *Drosophila *males, the mechanism(s) by which this is achieved remains controversial [33]. One model postulates that MOF, in association with other members of the MSL complex, binds to the hemizygous X-chromosome resulting in a two-fold increase in X-linked gene expression [34,35]. According to this model, MOF acetylates H4 on lysine 16 [H4Ac16] resulting in a relaxed chromatin configuration and a consequent elevation in gene expression. While our data indicate that MOF interacts with hemizygous copies of *copia*, the consequence is repression rather than elevation in expression. This suggests that the regulatory functions of *Drosophila *MOF may be context dependent.

The alternative "inverse regulator model" of dosage compensation postulates that hemizygosity of the male X-chromosome results is a genome-wide elevation in gene expression [19,36]. The significance of the sequestering of the MSL complex to the male X-chromosome in this model is two-fold. First removal of the MOF acetyltransferase from autosomes is postulated to attenuate the increased level of autosomal gene expression due to the X dosage affect. Secondly, the binding of the MSL complex prevents X-linked genes from responding to the elevated levels H4Ac16. The mechanism(s) underlying this second proposed function is unknown, although it has been demonstrated that the expression of at least some X-linked and autosomal genes are significantly elevated in male *mof *mutants [19,36]. This suggests that MOF alone or within the context of the MSL complex can act to repress expression of at least some X-linked genes. This finding is consistent with our observation that MOF represses *copia *expression in the hemizygous condition.

Since retrotransposons significantly predate the evolution of dosage compensation, our results suggest that the original function of MOF and perhaps other members of the MSL complex was to silence retrotransposons and that these functions were later co-opted in the evolution of dosage compensation in *Drosophila*.

## Conclusion

A growing body of evidence in both plants and animals indicates that epigenetic mechanisms originally evolved as a defense against transposable elements and were subsequently co-opted for a variety of cellular functions [37-39]. Our findings are consistent with this model and suggest that at least some of the mechanisms underlying dosage compensation in *Drosophila *may have their origins in processes originally evolved to defend against the mutagenic potential of transposable elements.

## Methods

### Fly strains

All mutant fly strains were obtained from the Bloomington Stock Center. Wild strain 194 was established from a collection in Athens (Georgia, USA) in June 2003. Crosses were performed at 25°C on yeast, cornmeal, molasses, and agar medium. Details of the *Drosophila *strains and genetic crosses used in the construction of flies/larvae used in this study are provided in the Additional file 1.

### Transgenic flies and CAT-assay

The full-length *copia *LTR-CAT construct described previously [40] was sub-cloned from *pCopiaCAT *into *Pst1-Xba1 *site of the *pCaSpeR *[41] transformation vector. Germ line transformation was performed by microinjection of 1 h embryos. Injected survivors were backcrossed to *w*^*1118 *^males or females and transformants were identified by eye color. Insertion sites were determined by *in situ *hybridization of pCAT plasmid (Promega) to polytene salivary gland chromosomes of third instar larvae. The probe was labeled with biotin (bio-dUTP) by nick-translation. Hybridization was detected using the Vestastain ABC kit (Vector Labs) and diaminobenzidine (Sigma). The transgene locations were determined according to standard maps of Lefevre [42].

CAT activity was measured by liquid scintillation counting (LSC) of CAT reaction products. Crude protein extract from 1 fly/larva was incubated in a reaction mix containing C-14 chloramphenicol and n-butyryl Coenzyme A (Promega). Eight to10 flies were assayed individually for each variant (strain). Results are presented in CAT units per fly. 1 CAT unit is defined as the conversion of 1 nmol acetyl coenzyme A to chloramphenicol/min at 37°C. Activity values are the average of 10 independent fly or larvae assays per strain. Means and standard deviations of CAT activity units were computed for each strain. Two tailed t-tests were used to test the significance.

### Northern analysis and RT-PCR

mRNA was isolated from 3rd instar larvae using the Oligotex Direct mRNA kit (Qiagen, Valencia, CA). RNA was electrophoresed through a 1% agarose-formaldehyde gel, transferred to nitrocellulose filter and hybridized as described [43]. The *copia *genomic clone DM5002 [43] was used as a probe. For loading control a *β*-tubulin probe [44] was included into the hybridization mix. Probes were labeled using a nick-translation kit (Roche).

For RT-PCR, mRNA was additionally treated with DNase (DNA-free kit, Ambion). cDNA was synthesized with Oligo(dT) primers, ThermoScript RT-PCR System (Invitrogen). PCR primers: *roo*-f 5'- TCC ATT CAA GGA TGT CAC C-3'; *roo*-r 5'- ATG CTT TTT CGG AGG CGT CC-3'; *1731*-f 5'-GCC ATT TGA ATA CAA GCA GCC TAC-3'; *1731*-r 5'- CGG GAT TAG CAG CAT CTG TGA AC-3'; *412*-f 5'- CAG TGT GCT AAG GCT TTG AAC CTA c-3'; *412*-r 5'- GAA CTT GGG CTT GTA TTT CTT CCA C-3'; *297*-f 5'- ATT GCC AGT GAC CAT CAA CCT C-3'; *297*-r 5'- TGC TAC CCC GTT TTT TGC TG-3'; *copia*-f 5'-GGG AAG AAG CCA TCA ATA CAG-3'; *copia*-r 5'-CAA ATA CTT CAA ACC AGC ATC-3'; *gypsy*-f 5'- CGT AAT AAG TGT GCG TTG AAT-3'; *gypsy*-r 5'- CGA CCT TAA CCT TTC TGT AGT-3'; β-tubulin-f 5'- CAA GGC TTC CAA CTC ACA CAC TC-3'; β-tubulin-r 5'- AGG TGG CGG ACA TCT TCA GAC-3'.

### Nuclear isolation and chromatin analyses

Nuclei were prepared from third instar larvae, such that chromatin structure is preserved as described previously [12]. Standard DNA extraction (chromatin structure not preserved) from the same stage larvae was carried out using proteinase K digestion overnight followed by phenol-chloroform extraction. Digestion of nuclei or DNA with *Apa*1 was performed at 37°C for 1 h. Reactions were stopped by heating at 70°C for 10 min. PCR were performed with the following primers: *copia*-f 5'-GGG AAG AAG CCA TCA ATA CAG-3' *copia*-r 5'-CAA ATA CTT CAA ACC AGC ATC-3'; CAT-r 5'-CAC CGT CTT TCA TTG CCA TAC G 3'(See Figure 1).

### Chromatin immunoprecipitation

Chromatin immunoprecipitation was performed on the *Drosophila melanogaster *Kc167 cell line (female, by criterion of dsx splicing) obtained from the *Drosophila *Genomics Resource Center. Cells were grown to a density of 2 × 10^*6 *^to 4 × 10^*6 *^cells/ml. EZ CHIPTM Chromatin Immunoprecipitation Kit from Upstate Inc. (Chicago) was used according to manufactures instructions. MOF, MSL-1 and MSL-3 antibodies [21] were provided by Dr. John Lucchesi (Emory University), and normal mouse IgG antibody (Upstate Inc., Chicago) was used as a nonspecific control.

The chromatin immunoprecipitation polymerase chain reactions were quantified using the 2^-ΔΔC^T method [45]. PCRs were performed with primers specific for the *copia *ULR (5' untranslated leader region; f 5'GCCCAGTCCATGCCTAATAA-3'; r 5'-GCCTTGTCCATTTTTCACTCA-3'), the *roX-1 *gene [23] (positive control; f 5'-GTCGAATTCGAAAAACACATTTACTAACAAATAA-3'; r 5'-GTCGAATTCCCCAAAGAAATCCACATAACAT-3') and the *Spt-4 *gene [22] (negative control; f 5'-CTCGTGGTATCTATGCCATTTCTG-3'; r 5'-TCCACGATTCTTCATGTCACGTA-3') in the presence of cyber green. Reactions were monitored on the DNA Engine Opticon 2 Continuous Fluorescence Detector. The Opticon Monitor 2 Software v2.01 was used to calculate the C_T _for each reaction following subtraction of the minimum over cycle range background and manually setting the threshold to the linear range of amplification. Triplicate polymerase chain reactions were performed for each antibody precipitation. The average of three polymerase chain reactions of no antibody (beads only) precipitations was subtracted from each polymerase chain reaction with antibody to generate three ΔC_T _values for each immunoprecipitation. The average of three polymerase chain reactions of the IgG immunoprecipitation (non-specific control) was subtracted from each ΔC_T _value to yield three ΔΔC_T _values for each immunoprecipitation normalized to IgG. The average and standard errors for the three 2^-ΔΔC^T values were plotted to reveal the average fold increase of antibody precipitation reactions over the no antibody precipitation reaction controls.

## Authors' contributions

LVM helped conceive the study, conducted the experiments and contributed to the data analysis; NJB contributed to the data analysis; JFMcD conceived the study, contributed to the data analysis and wrote the paper.

## Supplementary Material

Additional file 2 Table 1-5. CAT activity in larvae or adult flies hemizygous for the construct within a series of genetic backgrounds mutant for genes known to affect chromatin structure in *Drosophila*:*LOW/+, HDAC 1*^*326*^*/+, HDAC 1*^*328*^*/+, E(z)*^*28*^*/+, E(z)*^*61*^*/+, Psc25/+, Sxl*^*fl *^*/+*, presence of extra Y chromosome or absence of Y chromosome. Click here for file

Additional file 1 *Drosophila *strains and genetic crosses. *Drosophila *strains and genetic crosses used in the construction of flies/larvae used in this study Click here for file
