# Supplementary material for: LTR retrotransposons and the evolution of dosage compensation in Drosophila
Source: BMC Mol Biol. 2008 Jun 4;9:55. doi: 10.1186/1471-2199-9-55 (PMC2443393; doi:10.1186/1471-2199-9-55)
Supplement: Additional file 2 — Table 1-5. CAT activity in larvae or adult flies hemizygous for the construct within a series of genetic backgrounds mutant for genes known to affect chromatin structure in Drosophila:LOW/+, HDAC 1326/+, HDAC 1328/+, E(z)28/+, E(z)61/+, Psc25/+, Sxlfl /+, presence of extra Y chromosome or absence of Y chromosome. [file 1471-2199-9-55-S2.doc]

Additional files

Additional file 2
File format: Word document

Title:.  Table 1-5
Description: CAT activity in larvae or adult flies hemizygous for the construct within a series of genetic backgrounds mutant for genes known to affect chromatin structure in *Drosophila* :*LOW/+, HDAC 1^326^/+, HDAC 1^328^/+, E(z)28/+, E(z)61/+, Psc25/+, Sxl ^fl^ /+ ,* presence of extra Y chromosome or absence of Y chromosome.

**Table 1. Expression level of *copia* LTR-CAT in three stably transformed strains of *Drosophila melanogaster*  hemizygous for the construct and made wild type (SM5) or heterozygous for the mutant of the *LOW (lightening of white)* allele (See Supplemental Figure for strain construction).** The results indicate no significant effect of *LOW* on *copia* LTR-CAT.

| Strain | Position | ♀ Tr/SM5 | ♀ Tr/LOW | ♂ Tr/SM5 | ♂ Tr/LOW |
| --- | --- | --- | --- | --- | --- |
| 9-3 | 3L 80A | 0.85(0.09) | 0.73(0.10) | 0.62(0.05) | 0.64(0.12) |
| 9-4 | 2R 57B | 0.23(0.03) | 0.22(0.02) | 0.25(0.00) | 0.29(0.05) |
| 39-2 | X 5A | 0.19(0.02) | 0.21(0.02) | 0.73(0.05) | 0.73(0.10) |

**Table 2.** **Expression level of *copia* LTR-CAT in six stably transformed strains of *Drosophila melanogaster*  hemizygous for the construct and made wild type (TM3) or heterozygous the mutant for the *HDAC1^326^* or *HDAC1^328^* (*Histone Deacetylase*) alleles (See Supplemental Figure for strain construction).**  The results indicate a slight but significant decrease in *copia* LTR-CAT expression in strains mutant for the *HDAC1^326^* or *HDAC1^328^*  mutant.

| Genotype | 9-3  3L 80A | 9-4  2R 57B | 9-6  3L 76B | 14-1  X 16B | 14-2  4 102B | 39-2  X 5A |
| --- | --- | --- | --- | --- | --- | --- |
| ♀ Tr/TM3 | 1.60(0.27) | 0.27(0.09) | 0.70(0.11) | 0.25(0.05) | 0.28(0.05) | 0.24(0.03) |
| ♀ Tr/HDAC1^328^ | 0.97(0.16)* | 0.14(0.03)* | 0.46(0.12)* | 0.14(0.02)* | 0.19(0.03) | 0.16(0.03) |
| ♀ Tr/HDAC1^326^ | 0.88(0.15)* | 0.14(0.03)* | 0.43(0.06)* | 0.17(0.05)* | 0.15(0.02)* | 0.15(0.02)* |
| ♂ Tr/TM3 | 1.31(0.28) | 0.25(0.05) | 0.59(0.16) | 1.17(0.10) | 0.43(0.13) | 1.00(0.18) |
| ♂ Tr/HDAC1^328^ | 0.93(0.16) | 0.14(0.02)* | 0.31(0.05)* | 0.83(0.15) | 0.21(0.03)* | 0.64(0.05)* |
| ♂ Tr/HDAC1^326^ | 0.96(0.19) | 0.17(0.05)* | 0.30(0.03)* | 0.91(0.12) | 0.16(0.02)* | 0.75(0.21) |

* p < 0.01

**Table 3.** **Expression level of *copia* LTR-CAT in six stably transformed strains of *Drosophila melanogaster*  hemizygous for the construct and made wild type (TM3) or heterozygous mutant for the *E(z)^61^* *or E(z)^28^* (*Enhancer of zest*) or *Psc^25^* (*Posterior sex combs*) alleles (See Supplemental Figure for strain construction).** The results indicate a slight but significant decrease in *copia* LTR-CAT expression in the 9-3 transformed strain for the *E(z)^28^* or *Psc^25^* mutant.

| Genotype | 39-2 male | 39-2 female | 9-3 male | 9-3 female |
| --- | --- | --- | --- | --- |
| Tr/TM3 25C | 0.61 (0.13) | 0.15 (0.03) | 0.38 (0.09) | 0.54 (0.09) |
| Tr/E(z)^61^  29C | 0.42 (0.07) | 0.13 (0.05) | 0.43 (0.09) | 0.51 (0.09) |
| 25C | 0.55 (0.21) | 0.12 (0.02) | 0.38 (0.10) | 0.42 (0.08) |
| 18C | 0.72 (0.16) | 0.26 (0.11) | 0.42 (0.12) | 0.41 (0.07) |
| Tr/E(z)^28^  29C | 0.60 (0.14) | 0.09 (0.02) | 0.34 (0.07) | 0.34 (0.07)* |
| 25C | 0.58 (0.12) | 0.11 (0.01) | 0.33 (0.10) | 0.40 (0.12) |
| 18C | 0.63 (0.17) | 0.19 (0.04) | 0.29 (0.08) | 0.28 (0.06)* |
| Tr/Psc^25^ | 0.57 (0.04) | 0.10 (0.01) | 0.23 (0.03)* | 0.17 (0.03)* |

*p < 0.01

**Table 4.** **Expression level of *copia* LTR-CAT in three stably transformed strains of *Drosophila melanogaster*  hemizygous for the construct and made wild type (FM4) or heterozygous mutant for the *Sxl^fl^* (*Sex lethal*) allele (See Supplemental Figure for strain construction).** The results indicate no significant effect of *Sxl^fl^* on *copia* LTR-CAT.

| Genotype | 39-2 X 5A | 9-3 3L 80A | 9-4 2R 57B |
| --- | --- | --- | --- |
| Tr/FM4 male | NA | 0.83 (0.30) | 0.25 (0.03) |
| Tr/FM4 female | 0.016 (0.03) | 0.69 (0.09) | 0.19 (0.03) |
| Tr/Sxl male | NA | 0.79 (0.14) | 0.25 (0.05) |
| Tr/Sxl female | 0.16 (0.03) | 0.66 (0.11) | 0.22 (0.07) |

**Table 5.** **Expression level of *copia* LTR-CAT in two stably transformed strains of *Drosophila melanogaster*  hemizygous for the construct and wild type (XY or XX) or mutant (XO, XXY) for sex chromosome alleles.** The results indicate a slight but significant decrease in copia LTR-CAT expression in X/0 and XXY flies relative to controls (XY and XX respectively).

| Strain | Position | Male X/Y | Male X/O | Female X/X | Female X/X/Y |
| --- | --- | --- | --- | --- | --- |
| 14-1 | X 16B | 4.02 (0.32) | 3.17 (0.39)* | 0.91 (0.09) | 0.69 (0.04)* |
| 39-2 | X 5A | 1.06 (0.23) | 0.66 (0.12)* | 0.20 (0.04) | 0.13 (0.01)* |

*p<0.01
