# Supplementary material for: LTR retrotransposons and the evolution of dosage compensation in Drosophila
Source: BMC Mol Biol. 2008 Jun 4;9:55. doi: 10.1186/1471-2199-9-55 (PMC2443393; doi:10.1186/1471-2199-9-55)
Supplement: Additional file 1 — Drosophila strains and genetic crosses. Drosophila strains and genetic crosses used in the construction of flies/larvae used in this study [file 1471-2199-9-55-S1.doc]

Additional files
Additional file 1
File format: Word document

Title:.  *Drosophila* strains and genetic crosses
Description: *Drosophila* strains and genetic crosses used in the construction of flies/larvae used in this study

1. A *♀w^1118^/w^1118^*  +/+ *3^tr 9-3^*/*3^tr 9-3^ x ♂w^1118^/Y 2^nd9-4^/2^nd9-4^ +/+*

*♀w^1118^/w^1118^*  *2^nd9-4^*/+ *3^tr 9-3^*/*+*

*♂w^1118^/Y 2^nd9-4^/+ 3^tr 9-3^/+*

B *♀w^1118^/w^1118^*  +/+ *3^tr 9-3^*/*3^tr 9-3^ x ♂w^1118^/Y +/+ 3^tr 9-6-^/3^tr 9-6^*

*♀w^1118^/w^1118^*  *+/*+ *3^tr 9-3^*/*3^tr 9-6^*

*♂w^1118^/Y +/+ 3^tr 9-3^/3^tr 9-6^*

C *♀w^1118^/w^1118^*  +/+ *3^tr 9-3^*/*3^tr 9-3^ +/+ x ♂w^1118^/Y +/+ +/+ 4^th14-2^/4^th14-2^*

*♀w^1118^/w^1118^*  *+/*+ *3^tr 9-3^*/*+ 4^th14-2^/+*

*♂w^1118^/Y +/+ 3^tr 9-3^/+ 4^th14-2^/+*

D *♀w^1118^/w^1118^*  +/+ *3^tr 9-6^*/*3^tr 9-6^ x ♂w^1118^/Y 2^nd9-4^/2^nd9-4^ +/+*

*♀w^1118^/w^1118^*  *2^nd9-4^*/+ *3^tr 9-6^*/*+*

*♂w^1118^/Y 2^nd9-4^/+ 3^tr 9-6^/+*

E *♀w^1118^/w^1118^*  *2^nd9-4^/2^nd9-4^*  +/+ *+/+ x ♂w^1118^/Y +/+ +/+ 4^th14-2^/4^th14-2^*

*♀w^1118^/w^1118^*  *2^nd9-4^*/+ *+/*+ *4^th14-2^/+*

*♂w^1118^/Y 2^nd9-4^*/+  *+/+ 4^th14-2^/+*

F *♀w^1118^/w^1118^*  +/+ *3^tr 9-6^*/*3^tr 9-6^ +/+ x ♂w^1118^/Y +/+ +/+ 4^th14-2^/4^th14-2^*

*♀w^1118^/w^1118^*  *+/*+ *3^tr 9-6^*/*+ 4^th14-2^/+*

*♂w^1118^/Y +/+ 3^tr 9-6^/+ 4^th14-2^/+*

2. *♀y^1^mof^1^/Basc x ♂w^1118^/Y 3^tr^/3^tr^*

*↓*

*♂y^1^mof^1^/Y 3^tr^/+ ♂Basc/Y 3^tr^/+* (control)

3. *♀w^1118^/w^1118^ 3^tr^/3^tr^ x ♂ Low/SM5a*

*↓*

*♂ w^1118^/Y Low/+ 3^tr^/+ ♂ w^1118^/Y SM5a/+ 3^tr^/+*(control)

*♀w^1118^/X Low/+ 3^tr^/+ ♀w^1118^/X SM5a/+ 3^tr^/+*(control)

4. *♀w^1118^/w^1118^ 3^tr^/3^tr^ x ♂w^m4^/Y HDAC1^326^/TM3 Ser*

*↓*

*♂w^1118^/Y HDAC1^326^/3^tr^ ♂w^1118^/Y TM3 Ser/3^tr^*(control)

*♀w^1118^/X HDAC1^326^/3^tr^ ♀w^1118^/X TM3 Ser/3^tr^*(control)

5. *♀w^1118^/w^1118^ 3^tr^/3^tr^ x ♂w^m4^/Y HDAC1^328^/TM3 Sb Ser*

*↓*

*♂w^1118^/Y HDAC1^328^/3^tr^ ♂w^1118^/Y TM3 Sb Ser/3^tr^*(control)

*♀w^1118^/X HDAC1^328^/3^tr^ ♀w^1118^/X TM3 Sb Ser/3^tr^*(control)

6. *♀w^1118^/w^1118^ 3^tr^/3^tr^ x ♂E(z)^61^ e^11^/TM3 Sb Ser*

*↓*

*♂ w^1118^/Y 3^tr^/ E(z)^61^ e^11^ ♂ w^1118^/Y 3^tr^/ TM3 Sb Ser*(control)

*♀ w^1118^/X 3^tr^/ E(z)^61^ e^11^ ♀ w^1118^/X 3^tr^/ TM3 Sb Ser*(control)

7. *♀w^1118^/w^1118^ 3^tr^/3^tr^ x ♂E(z)^28^ st ts/TM3 Sb Ser*

*↓*

*♂ w^1118^/Y 3^tr^/ E(z)^28^ st ts ♂ w^1118^/Y 3^tr^/ TM3 Sb Ser*(control)

*♀ w^1118^/X 3^tr^/ E(z)^28^ st ts ♀ w^1118^/X 3^tr^/ TM3 Sb Ser*(control)

8. *♀w^1118^/w^1118^ 3^tr^/3^tr^  x ♂Psc^25^ ts red/y^+^ TM3 Sb e Ser*

*↓*

*♂w^1118^/Y 3^tr^/ Psc^25^ ts red ♂w^1118^/Y 3^tr^/y^+^ TM3 Sb e Ser* (control)

*♀w^1118^/X 3^tr^/ Psc^25^ ts red ♀w^1118^/X 3^tr^/y^+^ TM3 Sb e Ser* (control)

9.  *♀y Sxl^fls^ oc v f/FM6 x ♂w^1118^/Y 3^tr^/3^tr^*

*↓*

*♂y Sxl^fls^ oc v f/Y 3^tr^/+ ♂FM4/Y 3^tr^/+*(control)

*♀y Sxl^fls^ oc v f/X 3^tr^/+ ♀FM4/X 3^tr^/+*(control)

| **symbol** | **Description** |
| --- | --- |
| *2^nd9-4^* | Second chromosome with transgene insertion at 2R 57B |
| *3^tr9-3^* | Third chromosome with trnsgene insertion at 3L 80A |
| *3^tr9-6^* | Third chromosome with trnsgene insertion at 3L 75C |
| *4^th14-2^* | Forth chromosome with transgene insertion at 4 102B |
| *+* | Normal chromosome |
| *Basc* | Balancer X chromosome |
| *e^11^* | ebony |
| *E(z)* | Enhancer of zeste, |
| *f* | forked |
| *FM4* | Balancer X chromosome |
| *HDAC1* | Histone Deacetylase |
| *Low* | Lightning of white apricot. |
| *mof^1^* | males absent on the first |
| *oc* | ocelliless |
| *Psc^25^* | Posterior sex combs |
| *red* | red Malpighian tubules |
| *Sb* | Stubble |
| *Ser* | Serrate |
| *SM5a* | Balancer second chromosome |
| *st* | scarlet |
| *Sxl^fl^* | Sex lethal |
| *TM3* | Balancer third chromosome |
| *ts* | telescope |
| *v* | vermilion |
| *w^1118^* | white |
| *y^1^* | yellow |
